# Communication between general practitioners and medical specialists in the referral process: a cross-sectional survey in 34 countries

**DOI:** 10.1186/s12875-020-01124-x

**Published:** 2020-03-17

**Authors:** Giacomo Scaioli, Willemijn L. A. Schäfer, Wienke G. W. Boerma, Peter M. M. Spreeuwenberg, François G. Schellevis, Peter P. Groenewegen

**Affiliations:** 1 Department of Public Health Sciences, University of Turin, Piazza Polonia, 94, 10126 Torino, Italy; 2 Department of Surgery, Northwestern University, Feinberg School of Medicine, 633 N. St Clair Street, Chicago, IL 60611 USA; 3 NIVEL (Netherlands Institute for Health Services Research), PO box 1568, Utrecht, 3500BN The Netherlands; 4 Department of General Practice and Elderly Care Medicine, Amsterdam Public Health Research Institute, VU University Medical Center, Van der Boechorststraat 7, Amsterdam, 1081 BT the Netherlands; 5 Department of Sociology and Department of Human Geography, Utrecht University, P.O. Box 80.115, Utrecht, 3508 TC The Netherlands

**Keywords:** Continuity of care, Communication, Primary care, Secondary care, Referrals

## Abstract

**Background:**

The communication of relevant patient information between general practitioners (GPs) and medical specialists is important in order to avoid fragmentation of care thus achieving a higher quality of care and ensuring physicians’ and patients’ satisfaction. However, this communication is often not carried out properly. The objective of this study is to assess whether communication between GPs and medical specialists in the referral process is associated with the organisation of primary care within a country, the characteristics of the GPs, and the characteristics of the primary care practices themselves.

**Methods:**

An analysis of a cross-sectional survey among GPs in 34 countries was conducted. The odds ratios of the features that were expected to relate to higher rates of referral letters sent and communications fed back to GPs were calculated using ordered logistic multilevel models.

**Results:**

A total of 7183 GPs from 34 countries were surveyed. Variations between countries in referral letters sent and feedback communication received did occur. Little of the variance between countries could be explained. GPs stated that they send more referral letters, and receive more feedback communications from medical specialists, in countries where they act as gatekeepers, and when, in general, they interact more with specialists. GPs reported higher use of referral letters when they had a secretary and/or a nurse in their practice, used health information technologies, and had greater job satisfaction.

**Conclusions:**

There are large differences in communication between GPs and medical specialists. These differences can partly be explained by characteristics of the country, the GP and the primary care practice. Further studies should also take the organisation of secondary care into account.

## Background

Continuity of care is one of the key elements of modern healthcare systems [1–4]. An increasing number of patients with chronic diseases and multimorbidity [5] usually receive health care from both general practitioners (GPs) and several medical specialists. This highlights the need for a connection between all the health care events of each patient, in short that care is coordinated [4, 6]. Continuity of care contains three major dimensions. Firstly, there must be personal continuity, meaning that patients have a personal physician who knows and follows them in separate care settings. Second is team continuity, which entails that relevant patient information is communicated between physicians within a care setting. Finally, cross-boundary continuity is important, that is the communication of relevant patient information and cooperation between physicians from different settings [2, 3, 7–9]. Previous studies showed that high rates of communication between physicians were associated with better patient outcomes and greater satisfaction among both patients and physicians [10–13]. Low rates of communication seem to decrease the quality of care due to delayed diagnoses and treatments, repeated diagnostic examinations, increased rates of adverse events, and avoidable hospitalisations [10, 14–17].

Communication between physicians usually takes place when a patient is referred by one physician to another. As such, it represents one of the most important steps in the referral process itself [18]. As referrals usually occur from a GP to a medical specialist, [19] it is relevant to focus our attention especially on these professionals. Despite this relevance, the exchange of patient information between GPs and medical specialists through referral letters by GPs and feedback reports by medical specialists has been demonstrated to be performed inadequately [20]. Studies conducted in the USA showed that in only 50% of the cases medical specialists did receive information about patients referred by general paediatricians [11, 21]. Another study stated that 68% of the medical specialists do not receive information from GPs [14].

Research has shown that communication between primary and secondary care was better when GPs indicated that they had ‘adequate time’ during consultations with patients [14, 22]. In addition, the presence of a nurse in the practice and the use of health information technologies (HITs), and/or a greater integration between GPs and medical specialists, have shown to improve communication [21–24]. Nurses and HITs can help GPs in the production and the delivery of referral letters, and also in obtaining feedback from the specialists by chasing them up.

So far, studies on correlates of communication between primary and secondary care were performed in single countries. International studies on referrals, meanwhile, have not focused on this specific topic [25]. The organisation of primary care varies greatly between countries which might affect attitudes and practices of GPs and, therefore, rates of communication between GPs and medical specialists [26, 27]. By using data from 34 countries, our study investigates whether differences between countries in the organisation of their primary care systems are related to the rate of communication between primary and secondary care. We then assess whether characteristics of GPs and primary care practices are associated with this communication, independent of the organisation of the primary care system in which GPs operate.

**Fig. 1:**
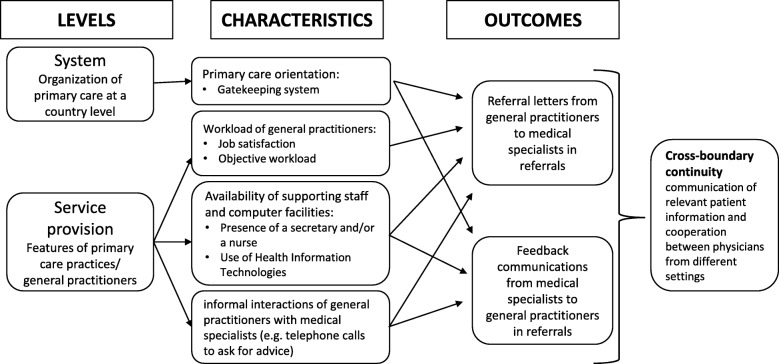
Visual model: features that potentially influence communication between GPs and medical specialists

The aim of our study is to assess what are the factors which explain the variation between countries in the rates of communication between GPs and medical specialists in referrals. Can they be explained by differences in how primary care is organised at the national level and by differences in the characteristics of GPS and primary care practices (Fig. 1)? We hypothesise that, in the referral process from a GP to a medical specialist:

1. Health care systems that are more orientated towards primary care, [26, 27] achieve higher rates of communication in both directions (referral letters sent and feedback communication received);
2. GPs who report a higher workload and lower satisfaction send fewer referral letters to medical specialists than those who reported a lower workload and greater job satisfaction;
3. GPs working with support staff and computer facilities, achieve higher rates of communication to, and from, medical specialists;
4. GPs who have more informal interactions with specialists have more communication to, and from, medical specialists.

## Methods

To assess the rates of communication between GPs and medical specialists in 34 countries we used data from the QUALICOPC study (Quality and Costs of Primary Care in Europe). In this study, GPs in 31 European countries were included (Austria, Belgium, Bulgaria, Cyprus, Czech Republic, Denmark, England, Estonia, Finland, Germany, Greece, Hungary, Iceland, Ireland, Italy, Latvia, Lithuania, Luxembourg, Malta, the Netherlands, Norway, Poland, Portugal, Romania, Slovakia, Slovenia, Spain, Sweden, Switzerland, the Former Yugoslav Republic of (FYR) Macedonia, Turkey) and three non-European countries (Australia, Canada, New Zealand). We aimed to achieve a nationally representative sample of GPs completing a questionnaire in each country (target size: *N* = 220; Cyprus, Iceland, Luxembourg, and Malta *N* = 80). Only one GP per practice or health centre participated in the study. Questionnaires were completed anonymously either on paper or electronically. Data collection took place between October 2011 and December 2013. Details about the study protocol, including the required sample sizes, the development of the questionnaire, and the participation of GPs have been published elsewhere [28–30].

### Measures

#### Communication

The indicators of communication between GPs and medical specialists were derived from the following questions:

- To what extent do you use referral letters (including details on provisional diagnosis and possible test results) when you refer patients to a medical specialist?
- To what extent do medical specialists inform you after they have finished the treatment or diagnostics of your patients?

Possible answers for the first question were “for all patients whom I refer”, “for most patients”, “for a minority of patients” and “seldom or never”. Possible answers for the second question were “(Almost) always”, “Usually”, “Occasionally”, and “Seldom or Never”.

#### Organisation of primary care

Whether or not a country is oriented towards primary care [26, 27] was established on the basis of the presence of a gatekeeping system. We derived these data in 30 countries from the Primary Health Care Activity Monitor (PHAMEU) study [31, 32]. Data from four countries (Australia, Canada, New Zealand and FYR Macedonia) were collected using the same methods as the PHAMEU study [33]. We considered countries with a gatekeeping system as being those with a full or partial (for a selection of referral physicians) gatekeeping system. We considered countries with no gatekeeping system as those with no mandatory gatekeeping, regardless of the presence of incentives for patients who contact the GP as a first step [32].

#### Workload and job satisfaction

Workload was defined objectively as ‘the amount of time that certain activities consume or the frequency with which certain activities take place’. [34] We used variables from the questionnaire that assessed the number of patient visits and telephone calls in a normal working day, and the number of weekly working hours. For job satisfaction, [34] we used a score based on six statements in the questionnaire. GPs were asked to agree, or disagree, with the following statements: ‘I feel that some parts of my work do not really make sense’, ‘My work still interests me as much as it ever did’, ‘My work is overloaded with unnecessary administrative detail’, ‘I have too much stress in my current job’, ‘Being a GP is a well-respected job’ and ‘in my work there is a good balance between effort and reward’. A higher score indicates greater job satisfaction. The internal consistency (Cronbach’s alpha), computed over the whole dataset, is 0.68.

#### Support staff and computer facilities

We included data about the availability of a secretary and/or a nurse [35] in the primary care practice (Yes/No). ‘Use of HIT in the referral process’ was derived from a question asking whether GPs use the computer to send referral letters to specialists (Yes/No).

#### Informal interactions

We calculated a score, based on the answers to the question ‘How often do you ask advice from the following medical specialists?’, the different medical specialists being: paediatrician, internist, gynaecologist, neurologist, dermatologist, geriatrician, psychiatrist, radiologist. A higher score indicates higher frequency of interactions.

### Statistical analysis

Firstly, we calculated the distribution of answers to the two communication questions. Next, we applied ordered logistic multilevel models to analyse the relationship between the two outcome variables and independent variables. Cases with missing data were excluded list-wise. Continuous variables were centred around the mean. We included two levels (country and GP) in our models. For each outcome variable, an initial model with background variables (age and gender of GPs, practice location and employment status of GPs) was estimated. Then, for the outcome ‘rate of referral letters’, we performed four cumulative models. We added variables as follows: 1) The objective workload and job satisfaction of GPs; 2) The availability of support staff and computer facilities; 3) The informal interactions of GPs with medical specialists; and 4) The organisation of primary care at a country level (gatekeeping system in place). For the outcome ‘reception of feedback communications from specialists’ we estimated just three models, since we did not hypothesise that the objective workload and job satisfaction of GPs might be associated with this outcome. We only present the final models in the tables. For each of the two outcome variables we calculated the intraclass correlation (ICC). We used π^2^/3 as approximation of the individual level variance [36]. We also calculated the percentage of reduction of variance in the final model at the country level. The total variance was approximated by using the linear predictor approach as described by Snijders and Bosker [36]. The level of statistical significance was set at *p* < 0.05. Descriptive and multilevel analysis were performed using Stata version 13.0.

## Results

### Rates of communication

A total of 7183 GPs completed the questionnaire (QUALICOPC database version 4.2). The median participation rate was 30% [30]. The background characteristics of GPs are described in Table 1.

**Table 1:** Characteristics of GPs, primary care practices, and countries included in the study (*N* = 7183)

*Characteristics of primary care practices and GPs*		*Median and interquartile range*	*Number of missing observations*
Gender (percentage of females)	52.5		28
Age (mean ± SD^a^)	50.3 ± 9.7	52 (43–58)	74
Practice location (percentages)			150
Big (inner)city	31.4		
Suburbs or small town	35.1		
Urban-rural or rural	33.5		
Employment status (percentages)			246
Salaried	35.0		
Self-employed	64.0		
Mixed	1		
Number of face-to face visits in a normal working day (mean ± SD^a^)	31.0 ± 16.0	28 (20–40)	49
Number of telephone calls in a normal working day (mean ± SD^a^)	8.2 ± 7.6	5 (3–10)	49
Job satisfaction (mean ± SD^a^)	2.5 ± 0.3	2,5 (2.2–2.8)	52
Availability of supporting staff (percentages):			137
Absence of secretary and nurse	7.4		
presence of a secretary or a nurse	43.1		
presence of both a secretary and a nurse	49.5		
Use of pc to send referral letters (percentage of ‘yes’)	71.0		292
Interactions between GPs and specialists (mean ± SD^a^)	1.7 ± 0.5	1.67 (1.2–2.0)	172
*Country characteristics*			
Gatekeeping system in place (percentage of ‘yes’)	55.9		0

^a^*SD* Standard Deviation

**Fig. 2:**
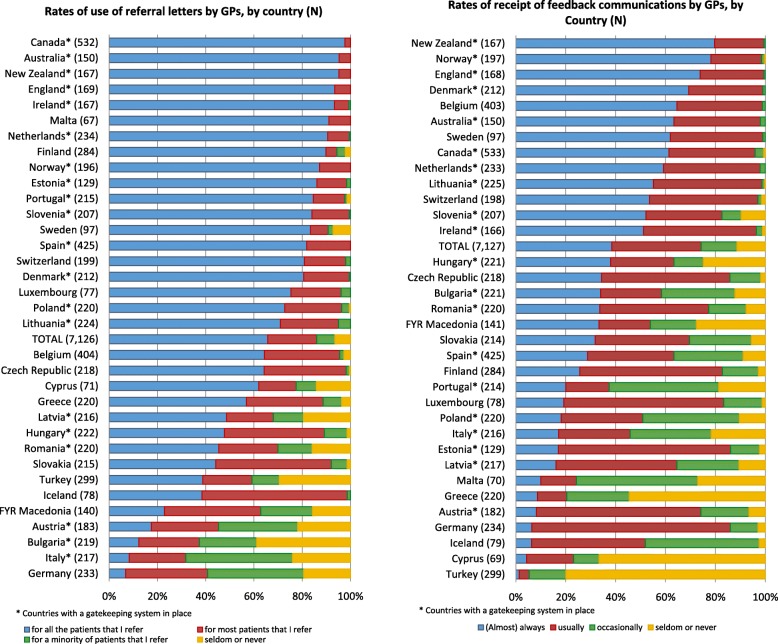
Rates of use of referral letters by GPs when referring a patient to a medical specialist, and of receipt of feedback communication by GPs after the referral, by country

Figure 2 shows the distributions of answers for the two communication variables by country. The majority (65.8%) indicated that they always send letters to a specialist when they refer a patient. There were, however, large differences between countries (e.g., 97.7% of GPs in Canada and only 6.9% in Germany). A total of 7.5% of GPs stated that they sent referral letters for a minority of the patients whom they refer. Furthermore, 6.5% stated that they ‘seldom or never’ send referral letters. In some countries (Denmark, Ireland, Malta, the Netherlands, Norway, Slovenia, Spain, England, Australia, Canada, New Zealand) almost all GPs send referral letters for all or most patients In other countries (Austria, Bulgaria, Germany, Italy), however, more than half of the GPs send referral letters seldom or never, or for a minority of patients whom they refer.

In total 38.4% of GPs ‘(almost) always’ receive feedback communications from specialists, with percentages that range from 79.6% in New Zealand to 1.3% in Turkey. Conversely, 11% of the GPs never receive feedback communications from specialists, with peaks in Turkey (79.9%) and in Cyprus (66.7%), and low percentages of GPs who never receive feedback in Belgium, Denmark, Lithuania, the Netherlands, Norway, Sweden, England, Australia, Canada, and New Zealand.

### Associated factors

Tables 2 and 3 show the results of the logistic multilevel models. In countries with a gatekeeping system, GPs reported higher rates of referral letters were sent. The reported use of referral letters was higher among GPs with a higher number of consultations per day and among GPs with a greater job satisfaction. The same holds for the presence of support staff and the use of computers. Moreover, GPs reported higher rates of referral letters were sent, when they have more informal interactions with specialists. The variance at country level is high (Intraclass correlation (ICC): 42% in the initial model). The proportion of the country level variance explained by this model is 7%.(Table 2) Comparing the third model (with all GP/practice characteristics) to the initial model, shows that the country level variance does not decrease with adding GP/practice characteristics. Hence, there are no marked composition effects. (not in table).

**Table 2:** Ordered logistic multilevel model of rates of referral letters sent by GPs in 34 countries

N_i_ = 34 N_j_ = 6580	Odds Ratio^a^ (CI)		p
*GP level*			
Number of face-to face visits in a normal working day	1.006	(1.001, 1.011)	0.009
Number of telephone calls in a normal working days	0.998	(0.990, 1.006)	0.499
Number of working hours per week	1.003	(0.997, 1.010)	0.297
Job satisfaction	1.22	(1.08, 1.39)	0.002
Availability of support staff (ref. = no secretary and no nurse)			
presence of a secretary or a nurse	1.33	(1.05, 1.70)	0.019
presence of both a secretary and a nurse	1.37	(1.05, 1.79)	0.020
Use of pc to send referral letters	1.78	(1.53, 2.09)	< 0.001
Interactions between GPs and specialists	1.45	(1.28, 1.63)	< 0.001
*Country level*			
Gatekeeping system in place	2.80	(1.01, 7.75)	0.048
Variance Country level (SE)	2.22	(0.56)	
Percentage of reduction of variance (Country level)^b^	7%		
Intraclass correlation (ICC)^c^	40%		

^a^ adjusted also for age and gender of GPs, practice location, and employment status of GPs

^b^ this percentage was calculated by using the variance at country level of the initial model (variance initial model =2.41, SE = 0.60)

^c^ the ICC of the initial model is 42%

N_i_ = number of countries

N_j_ = number of GPs

SE = standard error

CI = 95% confidence interval

**Table 3:** Ordered logistic multilevel model of rates of feedback communications received by GPs in 34 countries

N_i_ = 34 N_j_ = 6637a	Odds ratio^a^ (CI)		*P*
*GP level*			
Availability of support staff (ref. = no secretary and no nurse)			
presence of a secretary or a nurse	0.90	(0.71, 1.14)	0.375
presence of both a secretary and a nurse	0.94	(0.73, 1.21)	0.630
Use of pc to send referral letters	0.98	(0.85, 1.13)	0.786
Interactions between GPs and specialists	1.18	(1.06, 1.30)	0.002
*Country level*			
Gatekeeping system in place	3.60	(1.42, 9.13)	0.007
Variance Country level final model (SE)	1.86	(0.46)	
Percentage of reduction of variance (Country level)^b^	14%		
Intraclass correlation (ICC)^c^	36%		

^a^ adjusted also for age and gender of GPs, practice location, and employment status of GPs

^b^ this percentage was calculated by using the variance at a country level of the initial model (variance initial model =2.17, SE = 0.56

^c^ the ICC of the initial model is 39%

N_i_ = number of countries

N_j_ = number of GPs

SE = standard error

CI = 95% confidence interval

GPs report higher rates of feedback communications when they have higher rates of informal interactions with specialists. In countries with a gatekeeping system, GPs also report higher rates of feedback communication.(Table 3) Furthermore, for this outcome, variance between countries is high (ICC: 39% in the initial model). The proportion of country level variance explained by this model is 14%. Comparing the second model (with all GP/practice characteristics) to the initial model, shows that the country level variance does not decrease with adding GP/practice characteristics. Again, there are no marked composition effects. (not in table).

## Discussion

This study aimed to assess whether higher rates of communication between GPs and medical specialists during referrals are associated with the organisation of primary care at the country level and with characteristics of the primary care practices and GPs. Countries in which GPs play a gatekeeper role show higher rates of communication between GPs and medical specialists. A greater job satisfaction of GPs is positively related to the frequency of referral letters sent by GPs. A higher objective workload is associated with more referral letters being sent. The presence of support staff in the practice and the use of computers are positively related to the frequency of referral letters but not with the frequency of feedback communications. More informal interactions between GPs and specialists, for example through the use of telephone calls to ask for advice, is associated with higher frequencies of both referral letters and feedback communications.

Previously, a lower subjective workload (and therefore greater job satisfaction) was found to be associated with better performance by GPs and with greater patients’ satisfaction with their GPs [37]. The number of consultations could, possibly, not be a valid indicator of workload, because the time these consultations take might also be important. Moreover, GPs’ workloads are also determined by other tasks [38]. Our results are only partially consistent with a previous study conducted in the USA, in which the involvement of a nurse at the primary care practice was associated with higher rates of feedback communication received by GPs. But also the use of HITs was associated with higher reports of sending and receiving communications only by medical specialists alone, and not by GPs [22].

Our results confirm the need for GPs to play a pivotal role within health care organisations. Apparently, this is also recognised by specialists, who are more prone to sending feedback communications to GPs if they know that GPs have a central role in the management of care of their patients. This will be the case in countries with a gatekeeping system in place.

The results of this study can be useful for decision makers. Apparently, simple, modifiable features of GPs’ everyday practice, such as the use of a computer, the support of a secretary and/or a nurse, and a high number of interactions with specialists, may be helpful for the delivery of services of a better quality. In countries such as Belgium, Greece, and Italy, at least one quarter of the practices do not have a secretary or a nurse. In these countries, policies aimed at favouring the employment of such professionals might be promoted [39]. At the national and regional level decision makers can implement electronic patients’ health record systems [40]. These systems have shown to be useful in improving the share of patients’ information among physicians [23]. Decision makers may also play a role in promoting interactions between health care providers. Policies aimed at building a network that allows regular contacts between GPs and medical specialists seem to be useful in improving communication in both directions when a patient is referred. Interaction between different health care professionals has been associated with better health outcomes and lower avoidable hospitalisation [41]. Finally, policies aimed at increasing job satisfaction, for example by balancing efforts and rewards, and/or by increasing the awareness of GPs about the importance of their role in health care systems, can help in this regard [42].

The results of our study demonstrate that the gatekeeper role of GPs explains only a minor part of the variance at a country level in the communications between GPs and medical specialists. Therefore, there must be characteristics of the organisation of health care at a national level that were not included in our study, but which influence the delivery of referral letters and the receipt of feedback communication by GPs. It must be also noted that, in our study, primary care practices’ characteristics are not associated with receiving feedback communications from medical specialists. The receipt of these communications is more likely to depend upon medical specialists’ attitudes and the characteristics of the specialist medical setting [21, 22].

The main strength of this study is that it involves 34 countries, and thus allows estimates to be made of the association between the organisation of primary care at a national level and the outcomes of this study. The study also allows us to assess the GPs’ characteristics that are associated with communication between primary and secondary care, independent of the organisation of the primary care system in which GPs operate. Another strength is the use of multilevel regression analysis. This allows to examine simultaneously the effects of predictors at group- and at individual-levels, in order to account for the non-independence of observations within groups. It allows to examine both variation between individuals and between groups [43, 44]. With regard to the basic demographic characteristics age and gender, the study populations of GPs were representative of GPs in the respective countries [30]. Our study was conducted mainly in European countries and mostly among member states of the European Union. Three countries outside Europe participated in the study. Most of the countries participating were high income countries. This limits the generalisability of the results. We do not know whether the associations found, will also be valid in low and middle income countries and outside of Europe. The main limitation is that we have only data from GPs and, as explained above, we cannot estimate which characteristics of medical specialists involved in the referral process are associated with the delivery of referral letters and the receipt of feedback communications by GPs. Moreover, both of our outcome questions were addressed to GPs. Therefore, as we asked GPs about behaviours of medical specialists, there is a risk of reporting bias. It should also be mentioned that, in some countries, it is possible that patients self-refer to medical specialists without consulting a GP first. This may be the case particularly for certain categories of medical specialists, such as gynaecologists, internists, neurologists [32]. One of the two outcome questions included in this study (‘To what extent do medical specialists inform you after they have finished the treatment or diagnostics of your patients?’) might also include patients who self-referred. Our first outcome variable was based on the question of whether or not GPs send referral letters. We do not have additional information on the other means of communication from GPs to specialists in the referral process. We only mentioned referral letters in the question. Therefore, there might have been communication other than through a referral letter that we have missed. This limitation does not apply to the feedback information as the means of communication were not specified. Another limitation is that the study only evaluated primary care through data collected among GPs. In some countries there are also other providers of primary care who are not included in this study [28]. A final limitation is that we only evaluated the rates of referral letters sent and feedback communications received. We do not have information about the quality of these letters and the feedback nor about the referral process as a whole [11]. The QUALICOPC study was designed to cover a broad range of topics and not as a study into referral processes. We were therefore restricted in the nature and number of questions we could use. The quality of the referral process should be further explored in international research.

## Conclusion

There is a large variation among countries in the communication between GPs and medical specialists. The organisation of primary care at a country level, and specifically where there is a gatekeeping system in place, is associated with higher rates of communication. Characteristics of primary care practices and GPs are associated with rates of communication, independent of the health care system in which GPs operate. Moreover, this paper underlines the need for future studies to have a more comprehensive picture of the factors which, potentially, have a mutual relationship with the sharing of information between GPs and medical specialists in referrals.

## Data Availability

The datasets used and/or analysed during the current study are available from the corresponding author on reasonable request.

## Abbreviations

FYR Macedonia: Former Yugoslav Republic of Macedonia
GP(s): General practitioner(s)
HITs: Health information technologies
ICC: Intraclass correlation
PHAMEU: Primary Health Care Activity Monitor
QUALICOPC: Quality and Costs of Primary Care in Europe

## References

[1] World Health Organization (2008). The World Health Report 2008. Primary health care. Now More Than Ever.

[2] Kringos DS, Boerma WG, Hutchinson A (2010). The breadth of primary care: a systematic literature review of its core dimensions. BMC Health Serv Res.

[3] Uijen AA, Schers HJ, Schellevis FG (2012). How unique is continuity of care? A review of continuity and related concepts. Fam Pract.

[4] Haggerty JL, Reid RJ, Freeman GK (2003). Continuity of care: a multidisciplinary review. BMJ.

[5] Sinnige J, Korevaar JC, Westert GP (2015). Multimorbidity patterns in a primary care population aged 55 years and over. Fam Pract.

[6] Wagner EH, Sandhu N, Coleman K (2014). Improving care coordination in primary care. Med Care.

[7] Uijen AA, Heinst CW, Schellevis FG (2012). Measurement properties of questionnaires measuring continuity of care: a systematic review. PLoS One.

[8] Haggerty JL, Pineault R, Beaulieu MD (2008). Practice features associated with patient-reported accessibility, continuity, and coordination of primary health care. Ann Fam Med.

[9] Shang L, Waibel S, Thomson S (2013). Measuring care coordination: health system and patient perspectives. Report prepared for the Main Association of Austrian Social Security Institutions.

[10] Epstein RM (1995). Communication between primary care physicians and consultants. Arch Fam Med.

[11] Forrest CB, Glade GB, Baker AE (2000). Coordination of specialty referrals and physician satisfaction with referral care. Arch Pediatr Adolesc Med.

[12] Schoen C, Osborn R, Huynh PT (2006). On the front lines of care: primary care doctors' office systems, experiences, and views in seven countries. Health Aff.

[13] Anderson R, Barbara A, Feldman S (2007). What patients want: a content analysis of key qualities that influence patient satisfaction. J Med Pract Manage.

[14] Gandhi TK, Sittig DF, Franklin M (2000). Communication breakdown in the outpatient referral process. J Gen Intern Med.

[15] O'Malley AS, Cunningham PJ (2009). Patient experiences with coordination of care: the benefit of continuity and primary care physician as referral source. J Gen Intern Med.

[16] Garasen H, Johnsen R (2007). The quality of communication about older patients between hospital physicians and general practitioners: a panel study assessment. BMC Health Serv Res.

[17] Sutcliffe KM, Lewton E, Rosenthal MM (2004). Communication failures: an insidious contributor to medical mishaps. Acad Med.

[18] Foot CNC, Imison C (2010). The quality of GP diagnosis and referral.

[19] Coulter A, Roland M, Coulter A (1992). Does the referral system work?. Hospital referrals.

[20] Vermeir P, Vandijck D, Degroote S (2015). Communication in healthcare: a narrative review of the literature and practical recommendations. Int J Clin Pract.

[21] Stille CJ, McLaughlin TJ, Primack WA (2006). Determinants and impact of generalist-specialist communication about pediatric outpatient referrals. Pediatrics.

[22] O'Malley AS, Reschovsky JD (2011). Referral and consultation communication between primary care and specialist physicians: finding common ground. Arch Intern Med.

[23] Gandhi TK, Keating NL, Ditmore M, Henriksen K, Battles JB, Keyes MA (2008). Improving referral communication using a referral tool within an electronic medical record. Advances in patient safety: new directions and alternative approaches (Vol 3: performance and tools).

[24] Hsiao CJ, King J, Hing E (2015). The role of health information technology in care coordination in the United States. Med Care.

[25] Backer P, Crombie DL, van der Zee J (1990). The interface study. COMAC-HSR in collaboration with European General Practice Research Workshop. Occasional Paper 48.

[26] Boerma WG, van der Zee J, Fleming DM (1997). Service profiles of general practitioners in Europe. European GP task profile study. Br J Gen Pract.

[27] Boerma W (2006). Corrdination and integration in European primary care. In Saltman R, Rico, a, Boerma, W. primary care in the driver’s seat? Organizational reform in European primary care.

[28] Schafer WL, Boerma WG, Kringos DS (2011). QUALICOPC, a multi-country study evaluating quality, costs and equity in primary care. BMC Fam Pract.

[29] Schafer WL, Boerma WG, Kringos DS (2013). Measures of quality, costs and equity in primary health care instruments developed to analyse and compare primary care in 35 countries. Qual Prim Care.

[30] Groenewegen PP, Greß S, Schäfer W. General practitioners’ participation in a large, multi-country combined general practitioner – patient survey: recruitment procedures and participation rate. Int J Family Med. 2016:4929432. 10.1155/2016/4929432. 10.1155/2016/4929432 PMC4800081 27047689

[31] Kringos DS, Boerma WG, Bourgueil Y (2010). The European primary care monitor: structure, process and outcome indicators. BMC Fam Pract.

[32] Kringos DS, Boerma WG, Hutchinson A (2015). Building primary care in a changing Europe. WHO regional Office for Europe.

[33] Schafer WL, Boerma WG, Murante AM (2015). Assessing the potential for improvement of primary care in 34 countries: a cross-sectional survey. Bull World Health Organ.

[34] Groenewegen PP, Hutten JB (1991). Workload and job satisfaction among general practitioners: a review of the literature. Soc Sci Med.

[35] Groenewegen P, Heinemann S, Gress S (2015). Primary care practice composition in 34 countries. Health Policy.

[36] Snijders TAB, Bosker RJ (2012). Multilevel analysis: an introduction to basic and advanced multilevel modeling.

[37] Scheepers RA, Boerebach BC, Arah OA (2015). A systematic review of the impact of Physicians' occupational well-being on the quality of patient care. Int J Behav Med.

[38] Gottschalk A, Flocke SA (2005). Time spent in face-to-face patient care and work outside the examination room. Ann Fam Med.

[39] Ferre F, de Belvis AG, Valerio L (2014). Italy: health system review. Health systems in transition.

[40] Milieu Ltd, Time.lex. Overview of the nationals laws on electronic health records in EU Member States and their interaction with the provision of cross-boarder health services. Bruxelles; 2014. Available at http://ec.europa.eu/health/ehealth/docs/laws_report_recommendations_en.pdf. Accessed 16 Feb 2016.

[41] Martinez-Gonzalez NA, Berchtold P, Ullman K (2014). Integrated care programmes for adults with chronic conditions: a meta-review. Int J Qual Health Care.

[42] Siegrist J, Shackelton R, Link C (2010). Work stress of primary care physicians in the US, UK and German health care systems. Soc Sci Med.

[43] Diez-Roux AV (2000). Multilevel analysis in public health research. Annu Rev Public Health.

[44] Leyland AH, Groenewegen PP (2020). Multilevel modelling for public health and health services research: health in context.

[45] De Rosis S, Seghieri C. Basic ICT adoption and use by general practitioners: an analysis of primary care systems in 31 European countries. BMC Med Inform Decis Mak. 2015;15:70. 10.1186/s12911-015-0185-z PMC4546151 26296994

